# Cohort Profile Update: The British Regional Heart Study 1978–2014: 35 years follow-up of cardiovascular disease and ageing

**DOI:** 10.1093/ije/dyv141

**Published:** 2015-07-29

**Authors:** Lucy T Lennon, Sheena E Ramsay, Olia Papacosta, A Gerald Shaper, S Goya Wannamethee, Peter H Whincup

**Affiliations:** ^1^Department of Primary Care & Population Health, Institute of Epidemiology and Health Care, UCL Medical School, London, UK and; ^2^Population Health Research Institute, St George’s, University of London, London, UK

## Rationale for the new data collection

The earlier profile paper on the British Regional Heart Study (BRHS) was the first in the *International Journal of Epidemiology* series of cohort profiles, and it described 20 years of follow-up of the cohort of men aged 40–59 years from 24 towns across Britain.^1^ The BRHS was initiated to investigate regional variations in cardiovascular disease (CVD) mortality across Britain and to evaluate the potential importance of water hardness as a risk factor for CVD, a subject of considerable interest in the 1970s. The study also collected detailed information on a wide range of relevant exposures, health-related behaviours, social factors and biological risk markers in middle age. The initial follow-up focused exclusively on cardiovascular disease (particularly myocardial infarction, angina and stroke); subsequently other cardiovascular outcomes have been included (deep vein thrombosis, abdominal aortic aneurysm, pulmonary embolism, peripheral vascular disease, heart failure), together with diabetes and cancer. The previous profile paper described the follow-up of the cohort from middle age (40–59 years) until 60–79 years in 1998–2000, when a 20-year re-examination was carried out. Subsequent follow-up has continued to be based on 2-yearly primary care record reviews and annual update on mortality from the NHS Central Register, with postal questionnaires to study men in 2003, 2005 and 2007, 2010–12 (with a further re-examination) and 2014. From 2015, we plan to conduct annual primary care record reviews and annual questionnaires to the surviving participants.

### Physical ageing

Over the past 10 years, with the increasing age of the cohort from 60–79 years, opportunities have arisen to study new forms of CVD (particularly heart failure) and other forms of comorbidity and their relations to CVD risk, and to explore the scope for their prevention and management. A further examination of the cohort at age 71–92 years in 2010–12 provided an opportunity to extend the focus of the BRHS to improve our understanding of healthy ageing. The British Regional Heart Study is one of very few prospective epidemiological studies with detailed information on such a wide range of relevant exposures (health-related behaviours, social factors and biological risk markers) along with clinical measurements from middle age (40–59) through to 60–79 and 71–92 years, with extended follow-up for mortality and morbidity.

## New areas of research

An examination of the cohort at 71–92 years was carried out in 2010–12. This gave the opportunity to reassess anthropometry, body composition, blood pressure and lung function and also to collect new measures of cardiovascular ageing: physical function, objectively assessed physical activity, oral health measures and in particular quantitative assessments of vascular disease and vascular ageing including carotid intimal-medial thickness [CIMT], ankle brachial pressure index [ABPI] and carotid-femoral pulse wave velocity (PWV).

Preserving physical function, and reducing disability and frailty associated with CVD are crucial for the promotion of healthy ageing.^2^ At the examination we therefore extended our previous assessments of disability (all questionnaire-based) to include objective measures of physical function (including grip strength, walking speed and a chair stand test). This will enable us to study the impact of health-related behavioural and socioeconomic factors in the elderly not only on CVD prevention, but also on the prevention of disability and improvement of physical function.^2^^,^^3^ Cognitive function was also assessed by a self-completed ‘Test Your Memory’ survey.^4^

Another important aspect of this examination was to assess age-related changes in body composition, metabolic factors (adipocytes, insulin resistance and renal function through blood measurements) and inflammation, since these measures were also available from the previous examination at 60–79 years. Understanding age-related changes in these factors and their effects on CVD risk could provide novel insight into prevention of CVD and associated disability in the elderly.^2^^,^^4^^–^^6^

### Physical activity

The BRHS and other studies have reported that physical activity in later life is likely to have important effects on CVD and diabetes risks.^7^^,^^8^ However, the association between physical activity and CVD risk in later life has not been well quantified and the scientific basis for physical activity recommendations to prevent CVD in older people remains unclear. Moreover, most of the available data linking physical activity in later life with CVD risk is based on questionnaire assessment. This is less accurate and less detailed (particularly in defining the amount of time spent in activities at different levels of intensity) than objective methods for assessing physical activity which have recently become available. In addition, earlier studies have not taken account of sedentary behaviour, which is likely to contribute independently to CVD risk.^9^^–^^11^ Therefore, the follow-up at 71–92 years included objective physical activity assessment using a waist-worn activity monitor (the Actigraph GT3X) which was given to BRHS participants to wear for a 1-week period. These assessments of objectively measured physical activity have been repeated annually and will allow us to investigate the extent, determinants, health consequences and mechanisms of physical activity in older individuals.

### Oral health

There has been considerable interest in the influence of periodontal (gum) disease on CVD risk.^12^ This relates in part to shared risk factors (smoking, diabetes, age) and, recent wider interest in shared pathophysiological pathways (systemic inflammation and pathogens).^12^^–^^14^ Oral health problems in later life (such as tooth loss, poor oral function, xerostomia or dry mouth) also have significant implications on nutritional intake and quality of life.^15^ Thus, oral health is an important component of healthy ageing. The recent survey included an objective assessment of tooth count and periodontal disease along with questionnaire data on oral health outcomes (oral health-related quality of life, xerostomia, dental service use). Thus, the BRHS offers a unique opportunity to investigate the burden of oral health outcomes in the elderly and their impact on nutrition and quality of life, and to assess the role of risk factors associated with adverse oral health outcomes.

### Service in the armed forces

Alongside the re-examination of the surviving cohort, an initiative was taken to trace armed forces medical records for all participants of the cohort who had been in the armed forces either during the WWII or during National Service. These records were used to obtain information on anthropometry (height, weight) and oral health (number of teeth, dental decay) at 21 years. This will enable the BRHS to examine the relationship between anthropometric measurements and dental health in early adult life to CVD risk and vascular structure and function.

### Socioeconomic factors

The BRHS data is also being linked to routinely available data sources for additional information on socioeconomic factors as well as health outcomes. Data have been linked to obtain the ‘index of multiple deprivation’ for England, Wales and Scotland, an area-level socioeconomic measure. This will extend our previous work on socioeconomic inequalities to better understand the influence of area-level factors on health outcomes in later life. BRHS outcome data are also being extended to include data from hospital episode statistics (HES) to document individual comorbidities and hospitalizations among study participants.

## The cohort

**Figure 1.**
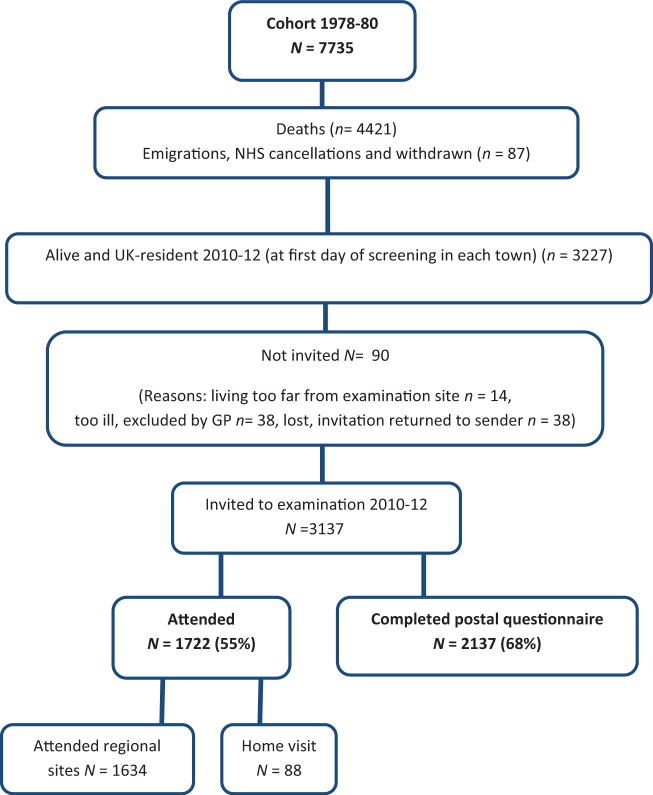
The attrition of the British Regional Heart Study cohort over 32 years from baseline until the new data collection at 71–92 years of age in 2010–12.

The British Regional Heart Study sample was drawn from 24 towns representing all major British regions and with populations between 50 000 and 100 000 in England, Scotland and Wales in 1974.^1^ Men aged 40–59 years were drawn at random from a single general practice register in each of these towns and were invited to participate. A socioeconomically representative cohort of 7735 men (78% response rate) was recruited and examined at baseline in 1978–80. The cohort has been followed up by a combination of primary care record reviews (morbidity), NHS Central Register flagging (mortality), periodic postal questionnaires (1992, 1996, 2003, 2005, 2007 and 2014) and re-examinations at ages 60–79 years (1998–2000) and 71–92 years (2010–12). The re-examinations were carried out in the original 24 towns where men were recruited at baseline. Participants who had migrated from their original town were offered a choice between examination either in their original town, another BRHS town of their choice or examination in London. Survey examinations were carried out in the original general practice, or where this was not possible a local health clinic or health authority or community premises were used. Study participants who were unable to attend the examination were offered a home visit with limited assessments. The cohort has been fairly stable, with little movement from the initial town of recruitment—less than 450 surviving men moved to a different town between baseline and 2010–12. A total of 1722 men attended the examination (55% response rate) at 71–92 years. A postal questionnaire was sent with the invitation to re-examination; this questionnaire was completed by 2137 men (68% response rate). Figure 1 describes the attrition of the cohort from baseline until the new data collection at 71–92 years.

## Measurements

At the most recent assessment men completed an additional questionnaire and attended a physical examination at a specified time between 0800 h and 1800 h after fasting for a minimum of 6 h; the assessments made are summarized in Table 1. Clinical measurements were carried out on each man by two trained research nurses and two vascular technicians in series. These included measurements of anthropometry, blood pressure, lung function and physical function, and dental assessments, resting electrocardiogram, bioelectric impedance analysis and fasting blood sample collection. Non-invasive cardiovascular measurements, including carotid ultrasound, carotid-femoral pulse wave velocity and ankle-brachial pressure index, were also taken. After the assessment, participants were provided with an Actigraph GT3X physical activity monitor to wear for a 1-week period. Following the initial assessment, participants have since been invited to have repeated annual assessments of physical activity with the Actigraph monitor sent by post; 1537 participants have provided further longitudinal physical activity data. New data linkages include information on comorbidities from the hospital episode statistics (HES), area-level factors from the index of multiple deprivation^16^ and the armed forces medical records for historical data on anthropometry and dental health.

**Table 1.** Additional data collected in the British Regional Heart Study at ages 71–92 years Clinical measurements were made on 7735 men (78% response) at baseline (1978–80), 4252 men (77% response) at 20-year re-examination (1998–2000); these have been described in details elsewhere.^1^ The 30-year re-examination (2010–12) was attended by 1722 men (55% response), using a similar protocol to the 20-year examination, and additional measurements are highlighted below

**Anthropometry**
Weight (Tanita BC418 Body Composition Analyser, electronic scale to the last complete 0.1 kg)
Mid-arm circumference (midpoint of the upper arm, between the acromial process and the lower tip of the olecranon recorded to the last completed millimetre)
**Physical function**
Grip strength (Jamar Hydraulic Hand Dynamometer Model J00105, measured thrice for each hand in kilograms)
Walking test (time taken, in seconds, to walk 3 metres at normal walking pace)
Chair stand test (time taken in seconds to stand up from a chair 5 times)
**Non-invasive cardiovascular measurements**
Carotid artery ultrasound performed using the Z.One Ultra ultrasound system (Zonare Medical Systems, Mountain View, CA) with a 5–10-mHz linear probe
Pulse wave velocity (PWV) measured using the Sphygmocor (Atcormedical, Australia) and the Vicorder (Skidmore Medical, Bristol UK)
Ankle brachial pressure index (ABPI) using the Vicorder device (Skidmore Medical, UK).
**Dental assessments**
Number of teeth, periodontal pocket, loss of attachment and bleeding on probing in six index teeth, one in each sextant
**Blood sample collection**
EDTA, citrate plasma and serum aliquots stored at −70°C
**Physical activity assessment—ongoing annual assessment**
Actigraph GT3X physical activity monitor worn for 1 week for objective physical activity measures along with a log diary
**Questionnaire—ongoing annual assessment**
Hearing, eyesight, sleep patterns, activities of daily living, dental health, memory, depression, local environment, medications, personal circumstances (marital status, accommodation) and diet. Test your memory self-completed questionnaire

## Recent findings

The BRHS has published extensively on many aspects of the epidemiology of cardiovascular disease, including risk factors (established and novel), risk prediction, trends, regional and social inequalities, and prevention. The newly collected data will allow investigation of new areas of research in later life with extended cardiovascular measures, novel inflammatory factors, physical function, physical activity, diet and oral health.

### Physical activity and sedentary behaviour in older men

Levels of physical activity are particularly low and sedentary behaviour particularly high among older men; only a small proportion of men achieve recommended physical activity levels.^21^ The markedly lower physical activity levels among older men substantially reflect lower levels of moderate and vigorous intensity physical activity, whereas light intensity physical activity is relatively spared; sedentary time (including long sedentary bouts) increase with age.^20^ Older people spend on average almost three-quarters of their day in sedentary behaviour, mostly accumulated in short bouts.^22^ Increasing the amount of moderate-intensity physical activity among older people could yield substantial health gains, though whether this activity needs to be in bouts of 10 min or more remains to be established.^21^ There has been considerable interest in the associations between falls, fear of falling and physical activity among older people. We have shown that a history of falls and, in particular, fear of falling are important barriers to physical activity among older people.^18^

### Frailty and CVD

A particular concern in older people is the development of frailty, defined as ‘a clinically recognizable state of increased vulnerability, resulting from ageing-associated decline in reserve and function across multiple physiological systems such that the ability to cope with everyday or acute stressors is compromised’.^23^ We have observed that frailty was cross-sectionally associated with increased risk of a range of cardiovascular factors (including obesity, low high-density lipo-protein cholesterol, hypertension, high heart rate, lower lung function, poor renal function) in older people; several of these cardiovascular factors were also raised or altered in those who were pre-frail.^19^ Moreover, these associations were independent of established CVD.^19^ The results highlight the burden of cardiovascular risk in the frail as well as pre-frail older populations, and thus the increased risk of CVD and its complications in frail older people. 

### Adiposity in early adult life and later chronic disease risk: evidence from armed forces records

Linkage of data from armed forces records has provided novel information on the association between high BMI at different points of the life course and cardiovascular and metabolic risk factors in later life.^17^ BMI in early adulthood had little influence on cardiovascular risk factors, but it was associated with later insulin resistance, suggesting some early patterning of diabetes risk.^17^ Further analyses have shown that BMI in later life is the dominant influence on cardiovascular and type 2 diabetes risk, although BMI in early adult life may have a small long-term effect on type 2 diabetes risk.^17^^,^^24^

Further areas of research are in progress. Key descriptive statistics for the cohort at 71–92 years are presented in Table 2. The results highlight the substantial burden of cardiovascular disease as well as comorbidities and disability in this elderly cohort.

**Table 2.** Characteristics of the British Regional Heart Study cohort at 71–92 years of age

**Characteristics from self-reported postal questionnaires (*N* = 2137)**	
Mean age in years (SD)	78.7 (4.8)
Manual social class *n* (%)	1003 (46.7)
Homeowner *n* (%)	1880 (89.2)
History of cardiovascular disease (angina, MI or stroke) *n* (%)	690 (32.7)
History of heart failure *n* (%)	49 (2.3)
History of deep vein thrombosis *n* (%)	69 (3.3)
History of diabetes *n* (%)	323 (15.4)
Fair/poor self-rated health *n* (%)	684 (32.6)
Hearing impairment *n* (%)	396 (19)
Fair/poor sleep quality *n* (%)	813 (38.8)
Mobility limitations *n* (%)	433 (26.2)
Current smokers *n* (%)	91 (4.2)
Moderate/heavy drinkers^a^ *n* (%)	263 (12.8)
Self-reported physical inactivity (none/occasional^b^) *n* (%)	880 (44)

**Measurements assessed through physical examination (*N* = 1722)**	
High blood pressure (≥160/90 mmHg or on antihypertensive treatment) *n* (%)	1255 (72.9)
High heart rate mean (SD)	67.1 (13.4)
Low HDL (<1.04 mmol/l) *n* (%)	230 (14)
High glucose (≥7 mmol/l) *n* (%)	156 (10.2)
Low haemoglobin (< 13 g/dl) *n* (%)	267 (16.6)
Low sodium (<138 mmol/l) *n* (%)	170 (10.4)
Obesity (BMI >30 kg/m^2^) *n* (%)	343 (20.1)
High waist circumference (>102 cm) *n* (%)	671 (39.3)
Mid-arm circumference (cm), mean(SD)	30.7 (2.9)
Triceps skinfold thickness (cm), mean(SD)	19.2 (7.9)
Grip strength (kg), mean (SD)	30.7 (10.1)
Walking speed (seconds), mean (SD)	3.6 (1.4)
Periodontal disease (>1–10% sites with loss of attachment > 5.5 mm) *n* (%)	456 (37)

MI, myocardial infarction; HDL, high-density lipoprotein; BMI, body mass index.

^a^Moderate/heavy drinkers defined as ≥ 16 units of alcohol/week.

^b^Physical activity scores assigned on the basis of frequency and type of activity such as walking, cycling and other sporting activities.^26^ The score comprises six groups: none, occasional, light, moderate, moderately vigorous and vigorous; none or occasional was classified as ‘inactive’.

## Collaborations

The BRHS is committed to maximizing the use of data collected over the past 37 years to advance scientific knowledge and has established strong collaborative links with a number of research groups—a few of these are mentioned below.

### British Womens Heart Health Study

British Womens Heart Health Study at: [http://www.lshtm.ac.uk/eph/ncde/research/bwhhs], which was set up in 1999–2001 as a parallel cohort of women, was designed to mirror the BRHS—See more at: [http://www.lshtm.ac.uk/eph/ncde/research/bwhhs]. This study is run by colleagues at the London School of Hygiene and Tropical Medicine. The Study aims to provide information about existing patterns of treatment of heart disease, and further the understanding of risk factors and disease prevention in women.

### Genetic consortiums—UCLEB and CARTA Consortium

UCLEB Consortium is coordinated by colleagues at the UCL Institute of Cardiovascular Science [https://www.ucl.ac.uk/cardiovascular/research/genetic-epidemiology-translational-cardiovascular-genomics], and investigates the genetic components for stratification, prediction, causal analysis and drug development in cardiometabolic diseases. The consortium for Causal Analysis Research in Tobacco and Alcohol (CARTA), coordinated by colleagues at the University of Bristol [http://www.bris.ac.uk/expsych/research/brain/targ/research/collaborations/carta] was established to investigate the causal effects of tobacco, alcohol and other lifestyle factors on health and sociodemographic outcomes using Mendelian randomization methods.

### Emerging Risk Factors Collaboration

The Emerging Risk Factors Collaboration (ERFC), coordinated by colleagues at the University of Cambridge [http://www.phpc.cam.ac.uk/ceu/research/erfc/], aims to determine to what extent the associations of several lipid and inflammatory markers with incident coronary heart disease outcomes are independent of possible confounding factors, and to what extent such markers (separately and in combination) provide incremental predictive value.

In addition, individual collaborators have provided expertise in specific scientific areas, including: Professor Julian Halcox, University of Swansea (vascular measurements); Professor Gordon Lowe, University of Glasgow (haemostatic and thrombotic markers); Professor Peter Macfarlane CBE, University of Glasgow (automated electrocardiography); Professor Naveed Sattar and Dr Paul Welsh, University of Glasgow (metabolic markers).

## Strengths and limitations

A major strength of the BRHS is that it is a socioeconomically and geographically representative sample of middle-aged and older men from across Britain.^1^ The cohort has benefited from high response rates throughout the follow-up, with near complete follow-up (>98%) for mortality and morbidity. Data collection and recording in the Study have been maintained to a very high standard since baseline. The wealth of data, including objective measurements from middle age, makes it a unique study to investigate determinants of cardiovascular disease and related health outcomes in the elderly; the extended data collection at 71–92 years allows the opportunity to research CVD-related aspects of healthy ageing.

Limitations of the BRHS include the limitation to men (although a parallel British Women’s Heart and Health Study^25^ was established in 1999) and the lack of representation from ethnic minority groups, which limits generalizability of findings to non-White British populations. The Study also avoided inner city populations and towns with high mobility. However, this has enabled the Study to have a stable cohort with high response rates.

## Data availability

Further details of the Study along with questionnaires, data collection forms and publications can be found on [http://www.ucl.ac.uk/pcph/research-groups-themes/brhs-pub]. The collection and management of data over the past 36 years of the BRHS have been made possible through grant funding from UK government agencies and charities. We welcome proposals for collaborative projects. For general data sharing enquiries, please contact Lucy Lennon [l.lennon@ucl.ac.uk].

British Regional Heart Study Update in a nutshell

- The British Regional Heart Study, a cohort of 7735 men aged 40–59, was set up in 1978–80 to investigate regional variations in cardiovascular disease (CVD) mortality across Britain.
- Opportunities for new research on CVD and related outcomes that affect healthy ageing are provided by 35 years of follow-up. New linkages enable examination of associations between early adult life measures and CVD risk, and vascular structure and function in old age.
- The most recent clinical examination in 2010–12 included 1722 men aged 71–92 years.
- In addition to a DNA databank, new measures include physical function, objective physical activity, oral health and quantitative assessments of vascular disease and vascular ageing. New data linkages provide information on comorbidities, area-level exposures and historical data on anthropometry and dental health.
- The British Regional Heart Study has several existing collaborations. For new collaborative projects and enquiries about data sharing please contact Lucy Lennon [l.lennon@ucl.ac.uk].

## Funding

The Study would not have been possible without the substantial funding we have received from the British Heart Foundation [since 2009, programme grants (RG/08/013/25942 and RG/13/16/30528)and project grants (PG/09/024,PG/13/41/30304 and PG/13/86/30546)]. SR is funded by a UK MRC Fellowship (G1002391). We are also grateful for funding from other funding bodies including the Department of Health, MRC, Diabetes UK and NIHR.
